# EpiVisR: exploratory data analysis and visualization in epigenome-wide association analyses

**DOI:** 10.1186/s12859-022-04836-2

**Published:** 2022-07-23

**Authors:** Stefan Röder, Gunda Herberth, Ana C. Zenclussen, Mario Bauer

**Affiliations:** 1 Department of Environmental Immunology, Helmholtz Centre for Environmental Research – UFZ, Leipzig, Germany; 2 Perinatal Research Group, Saxonian Incubator for Clinical Translation (SIKT), Medical Faculty, Leipzig University, Leipzig, Germany

**Keywords:** Visualization, EWAS, DNAm, Profile plot, Shiny application

## Abstract

**Background:**

With the widespread availability of microarray technology for epigenetic research, methods for calling differentially methylated probes or differentially methylated regions have become effective tools to analyze this type of data. Furthermore, visualization is usually employed for quality check of results and for further insights. Expert knowledge is required to leverage capabilities of these methods. To overcome this limitation and make visualization in epigenetic research available to the public, we designed EpiVisR.

**Results:**

The EpiVisR tool allows to select and visualize combinations of traits (i.e., concentrations of chemical compounds) and differentially methylated probes/regions. It supports various modes of enriched presentation to get the most knowledge out of existing data: (1) enriched Manhattan plot and enriched volcano plot for selection of probes, (2) trait-methylation plot for visualization of selected trait values against methylation values, (3) methylation profile plot for visualization of a selected range of probes against selected trait values as well as, (4) correlation profile plot for selection and visualization of further probes that are correlated to the selected probe. EpiVisR additionally allows exporting selected data to external tools for tasks such as network analysis.

**Conclusion:**

The key advantage of EpiVisR is the annotation of data in the enriched plots (and tied tables) as well as linking to external data sources for further integrated data analysis. Using the EpiVisR approach will allow users to integrate data from traits with epigenetic analyses that are connected by belonging to the same individuals. Merging data from various data sources among the same cohort and visualizing them will enable users to gain more insights from existing data.

## Background

DNA methylation has been demonstrated to be highly dynamic and essential for many biological processes. The importance of DNA methylation on biological processes has been shown for gene regulation and cell differentiation amongst others [1, 2]. With the advent of microarray technology in epigenetic research, researchers become supported with a deep and comprehensive overview on changes in DNA methylation applicable for epigenome-wide association analysis (EWAS). The Illumina Infinium HumanMethylation450 BeadChip (GPL13534 platform) became widely used to measure DNA methylation in roughly 485.000 CpG (cytosine followed by guanine nucleotide) sites [3]. Using the later developed Illumina MethylationEPIC BeadChip (EPIC array, GPL23976 platform) the coverage was expanded to about 850.000 CpG locations [4, 5].

A lot of EWAS have been performed using these techniques, which results in the increased availability of epigenetic datasets [6–12]. Usually, these results become reported and documented as differentially methylated CpG related to a certain trait [12, 13]. In environmental sciences dealing with the exposome [14], a common approach is based on searching for an association between (genome-wide) DNA methylation changes and the internal load on various chemicals (multi-trait analyses). Such an approach evokes a large number of results that have to be screened thoroughly. Common methods applied after calculating regression models are mainly based on filtering the results from corrected p-values. Large scale databases that collect and analyze such data exist [15]; they also allow enrichment as well as network analyses. In addition, approaches based on visualization by heatmap, such as *Complex Heatmap,* are in use [16, 17].

Many R based computational tools for analysis of DNA methylation data have been developed. An overview of existing toolsets can be found in [10]. In recent years online toolsets like EWAS Atlas [12], EWAS Data Hub [18] or EWAS Open Platform [15] emerged. They enable new interaction methods with already existing data, but lack specific interactive annotation. EpiVisR was conceived and developed to fill this gap.

**Fig. 1:**
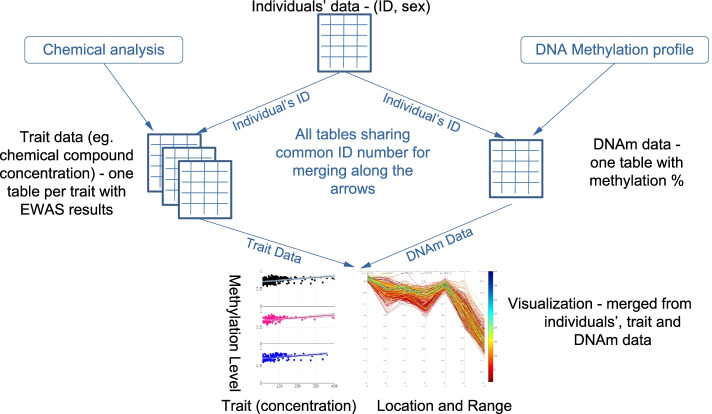
Relationship of trait, DNAm and EWAS analyses; each DNAm probe is available for many individuals as well as each trait; this allows merging of DNAm data with trait data by individuals Id as key attribute; visualization described here is based on this merge step

Deeper analysis of results found in EWAS can be tedious and time consuming. To get a faster overview about the biological importance of significant findings in EWAS. We propose EpiVisR, a toolset that integrates visualization as well as annotation and linkage to external data sources for further analyses. It enables the users to inspect results found in an a priori regression process (i.e. from meffil package [19]) visually. The visualization described here relies on individuals’ data from three domains (individuals’ data, trait data, DNAm data) as shown in Fig. 1.

The individuals table of the dataset in use (shown in Fig. 1) provides key information for joining data as well as information for stratification by sex. Trait data becomes merged to DNAm data to show up in various types of visualization. DNAm data itself is shown in the annotated Manhattan and volcano plots of EpiVisR.

## Implementation

EpiVisR provides a visualization module that shows data calculated by a prior calculation step. Due to heterogeneity of the data to be analyzed, the calculation step is implemented outside the EpiVisR package. Therefore, it can be easily adopted to the variable structure needed. Most notable here is the adjustment for cell type proportions as described in [5] and [20]. Also, adjustment for DNA methylation affecting the subject’s constitutions such as sex, gestational age, ethnicity, and obesity [21] should be taken into account.

The EWAS model calculation does not necessarily need a visual interface. We recommend using a software package such as *meffil* for model calculation [19]. The calculation module should provide results files with < P_VAL > , < Beta > , and < DeltaMeth > columns for each CpG. The *β*-value here is defined as

$$\beta = \frac{intensity\;methylated}{{intensity\;methylated + intensity\;unmethylated}}$$

for each CpG site [22]. Delta methylation is defined as the difference of methylation between highest and lowest found *β*-value at each CpG site:

$$\Delta_{methylation} = \max \left( \beta \right) - \min \left( \beta \right)$$

Other definitions for delta methylation (e.g. IQR) might be feasible and depend on the research question as well as on the used toolset for EWAS model calculation. Results should be stored as one file per trait. These files are then used in the visualization module: In a first step, all results from a certain folder are read into a data frame. Minimum as well as maximum values for all descriptive metrics are calculated. These aggregated values can be used for a first selection of trait under investigation out of a possible long list of traits.

Because of the static nature of the results, we implemented a caching strategy. Therefore it is not necessary to load .csv files over and over again. Instead, the data frame with all result summaries is stored in a separate .rds file for later and faster reuse.

EpiVisR was developed as web app on top of the shiny web application framework version 1.7.1 [23]. We used a modularized approach implementing shiny modules for easier maintaining, reuse, and future improvement. Even though the shiny framework offers the concept of reactive programming with seamless updates of related objects, we implemented EpiVisR in an event-driven approach. Otherwise, the users would have to wait at certain processing steps due to large amounts of data that have to be loaded between those processing steps. In consequence, every loading process must be started explicitly by the users.

Data tables in the user interface (UI) were built using the shiny DT module [24]. All plots are built on top of the widely used plotly framework [25]. Therefore, it is possible to drag, to move, and to zoom within the plots as well as to export these plots.

EpiVisR is controlled by a configuration file < config.yml > , which is stored in the same folder as the R scripts for visualization. The description of configuration parameters is included in the configuration file itself. Details on how to store data and how to set config file are given in the package vignette on GitHub (see the section “Availability and requirements”).

EpiVisR subdivides into several components: each component containing a UI as well as the server logic in one R file:

- < inputTrait.R > for selection of the folder containing the result files and for selection of trait under investigation;
-  < plotManhattanVolcano.R > for visualization of enriched Manhattan and volcano plots as well as for the selection of differentially methylated probes. Gene names as well as further genomic features of probes are annotated to probes in plots and tables. These gene names were annotated from the *meffil* R package [19] (where available) and become merged together with delta methylation values as well as p-values from the scenario of the currently analyzed trait under investigation;
- < plotTraitDNAm.R > for showing the relationship between trait under investigation and DNAm, stratified by sex;
- < plotDNAmProfile.R > for visualization of the genomic region around the differentially methylated probe;
- < DTCorrelatingProbes.R > for identification and plotting of probes somewhere on the epigenome that correspond to the probe currently under investigation;
- < server.R > containing and referring to the module’s server components;
- < ui.R > containing and referring to the module’s UI components;
- < util.R > containing several service functions not relating to a particular module.

EpiVisR works with current versions of Firefox, Edge and RStudio integrated browser. It can be installed from GitHub into an interactive R environment (RStudio) using devtools: devtools::install_github("steroe/EpiVisR").

## Results and discussion

Depending on the original research question, various formats are used in different EWAS databases. To distinguish between important and irrelevant CpG (probe) locations, at least a p-value as well as a methylation difference or methylation range (depending on the scale level of the trait under investigation) should be available in the dataset under investigation.

We propose the use of delta methylation values for differentially methylated probes (DMP) in addition to usually reported β values from regression models [21]. They allow selecting candidate probes with a methylation difference above a certain threshold. This enables further validation of findings by other methods than microarray technology (e.g., pyro sequencing). EpiVisR recognizes these attributes from their column labels: < P_VAL > , < Beta > , and < DeltaMeth > .

Example data was drawn from the LiNA cohort study [26, 27] and anonymized afterwards.

### Origin of data, preprocessing, and quality checking

Results from EWAS are usually generated by adjusted regression models and shared in long tables with probeIDs, p-values and delta methylation β for each trait under investigation that was analyzed. Thus, all common preprocessing steps to generate these β values (using *meffil* [19] or similar packages) were done in prior steps. EpiVisR starts with a table containing probeIDs, delta methylation values, and p-values. Unusual small p-values (near lower bound of the float data type in IEEE 754, the technical standard for floating-point computation [28, 29]) can be excluded [18]. Whether this is necessary depends on which system delivered the p-values from regression models. The same applies for models with small case counts (n). Both might be not reliable and therefore omitted.

EpiVisR can exclude multimodal probes from further processing. This works based on a pre-defined list of those probes, which is stored in a file named < MultiModProbesFileName > and referenced in the config file.

All selected probes are internally annotated with information provided by the *meffil* package [19]. This annotation is used in all visualization steps for annotation with gene symbols as well as other genomic features available from *meffil*.

### Selection of traits

**Fig. 2:**
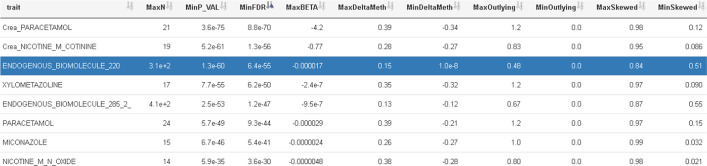
Trait selection using a list of filtering criteria; most important are the columns p-value and delta methylation; graph based scagnostics measures are also available

EpiVisR is able to process folders with results from many traits. They can be ordered by various measures such as the p-value, delta methylation, and other graph-based measures. The scatter plot of trait vs. methylation is available at least in the memory of software during runtime while building the regression models during the screening process. From this scatter image it is easy to calculate graph-based measures by using the scagnostics method [30, 31]. If these measures were calculated during the screening process, they can be used to filter and select traits for visual inspection. In case of a huge number of significant findings, these graph-based measures allow filtering for a further criterion before visual inspection of the results. Besides, measures such as the p-value or delta methylation can serve as selection criteria (Fig. 2).

### Visualization

EpiVisR has a user-friendly design to support the users in finding new insights. It offers the flexibility to use various types of visualization, supporting plots, and showing the relationship of a certain trait under investigation to a single CpG methylation as well as the methylation profile over a certain genomic region. The plots shown here use a data set which was distorted by batch effects for demonstration purposes.

**Fig. 3:**
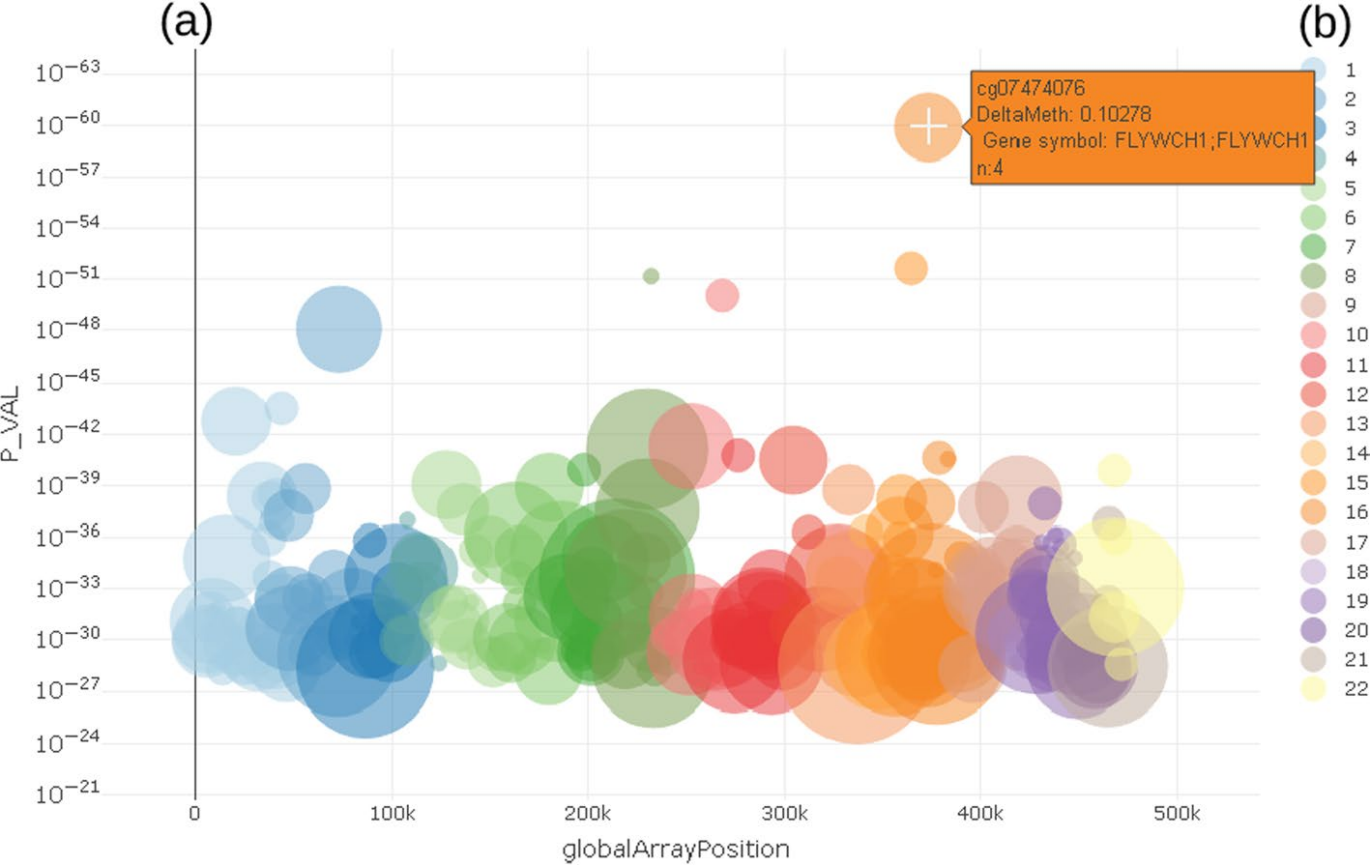
CpG selection using enriched Manhattan plot **a** showing p-values of differentially methylated CpG in relation to location on genomic region (chromosome). Color coding **b** shows chromosome location of CpG
(ii) In the enriched volcano plot [34] (Fig. 4) the -log10 p-value (P_VAL) of the regression model for a trait is shown against the delta methylation measure (DeltaMeth), provided by prior screening algorithm. Exemplarily, Fig. 4 shows a greater variance in hyper-methylated probes (DeltaMeth > 0) than hypo-methylated ones (DeltaMeth < 0), which is represented by a larger group of CpG on the right-hand side of the enriched volcano plot.

(i) The enriched Manhattan plot (Fig. 3) shows the -log10 p-value (P_VAL) of the regression model for a trait against the location of CpG on the microarray (globalArrayPosition) (Fig. 3). Each dot represents a CpG. The color coding visualizes the chromosome a certain CpG belongs to. To show and select only most important CpG locations, it is possible to limit the number CpG by % of top ranked p-values. Compared to a classical Manhattan plot [32, 33] the enrichment allows to identify important CpG in terms of prior knowledge.

**Fig. 4:**
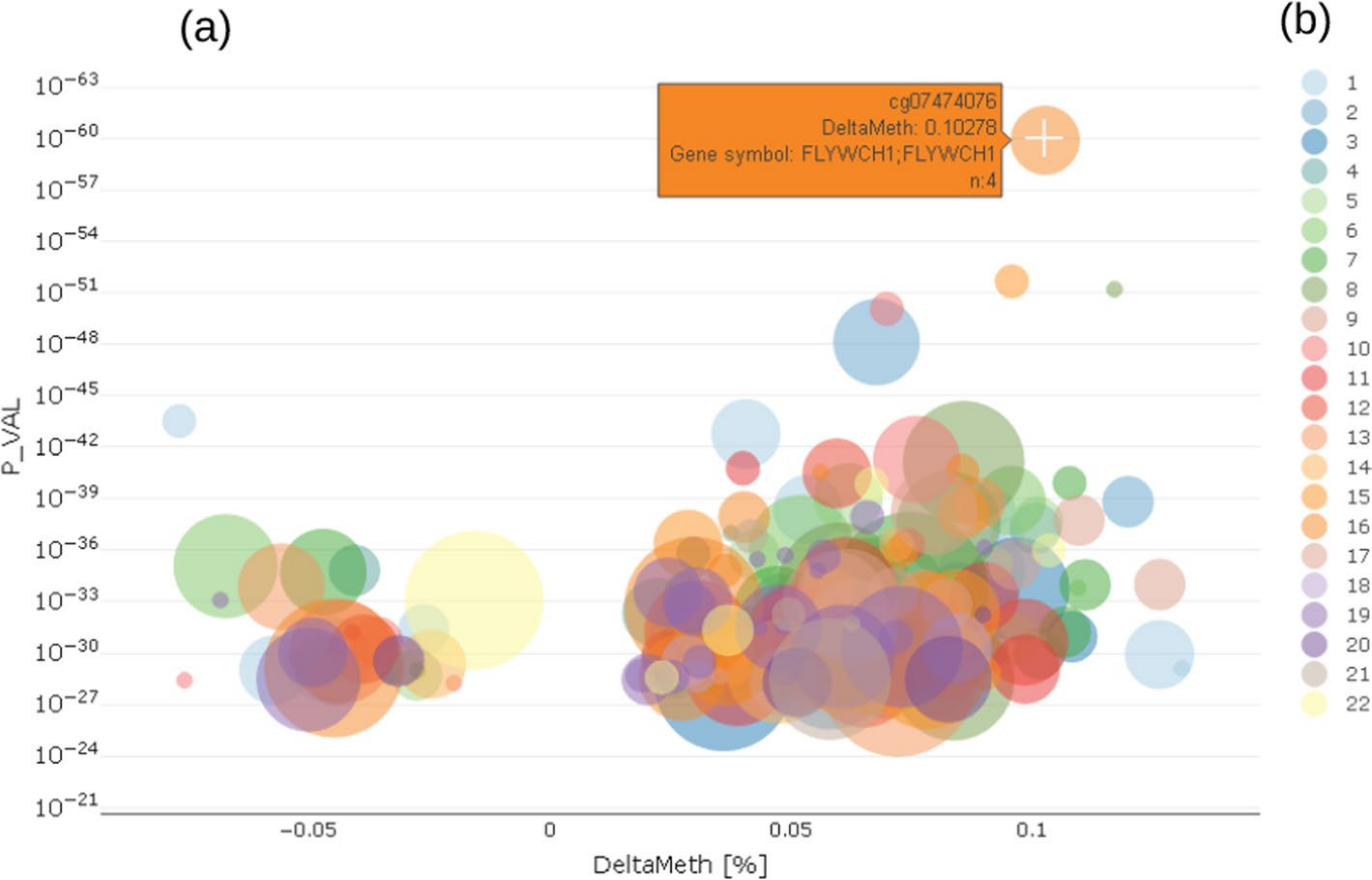
CpG selection using enriched volcano plot **a** showing relationship between p-values of differentially methylated CpG and delta methylation of those CpG; left side negative deltas, right side positive deltas; color coding **b** shows chromosome location of CpG
(iii) Both the Manhattan and the volcano plot can be enriched with further information, shown here by the size of the dots. This can be used to show the number of already known effects on a certain probe from an annotation table in order to decide more easily which effects are worth deeper inspection. In the examples shown here, we used data from the MRC-IEU EWAS catalog [35], showing the number of prior findings concerning a certain probe visualized by the diameter of each dot. From either the Manhattan or volcano plot, the users can select a single probe for deeper inspection of the relationship between the probe and trait under investigation by clicking on a certain dot (probe).
  The example shown in Fig. 3 highlights cg07474076, which is located on chr16 in gene FLYWCH1 and has n = 4 prior reports in the MRC-IEU catalog. Chromosome coordinates as well as further biological relevant information can be obtained from a table tied with the plots, which holds annotated information as text.
(iv) In a trait-methylation plot, the range of values in the trait under investigation vs. methylation at a certain CpG location can be shown. Lines represent the trend of trait – CpG relationship together with confidence interval as shadows. The trait-methylation plot can additionally be stratified to other attributes such as < gender > , which are stored in the traits table (Fig. 5). An additional horizontal violin plot shown below the trait-methylation plot visualizes the distribution of sex-stratified trait under investigation.

**Fig. 5:**
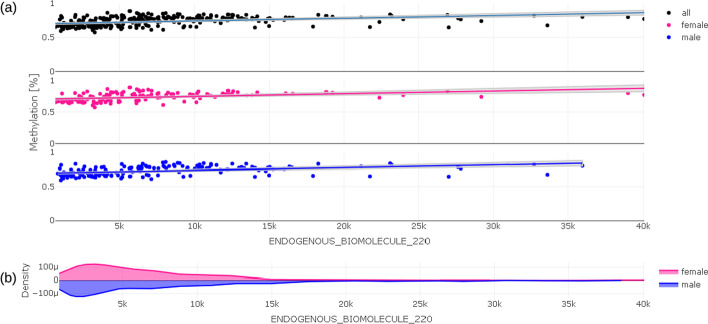
Trait-methylation plot (**a**) showing relationship between delta methylation and value of trait under investigation for all individuals (black), for females (pink), for males (blue); Distribution of values of trait under investigation is given in violin plot (**b**) below
(v) The methylation profile shows the methylation of all samples (connected by straight lines) covered by the EWAS on a single selected CpG in the center of the plot and its neighboring CpG (Fig. 6). The value (concentration) of the selected trait under investigation (e.g., chemical compound) is shown on a color scale to the right (c). The length of the visualization window can be adjusted according to the selected probe by using the input bar (a) above the plot (b). Distribution of values is visualized using the density plot (d). Most notably in this example is the clearly visible separation of values with high concentration values in trait under investigation (blueish/greenish lines) from the lower concentrations (reddish/ yellowish lines) over a range of three CpGs from the 3rd to the 5th probe in Fig. 6.

**Fig. 6:**
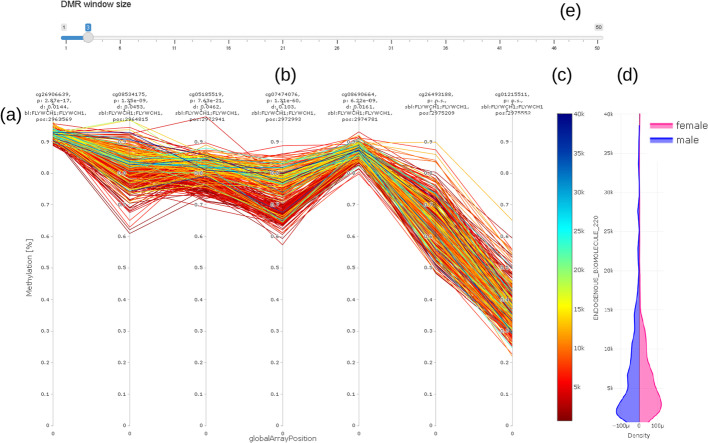
Methylation (DNAm) profile plot (**a**) showing methylation level (y axis) for each individual over a certain genomic range (globalArrayPosition on x axis) with selected CpG in the center (**b**); value (concentration) of trait under investigation is color coded (**c**) (red color for low concentration, blue for high concentration); violin plot (**d**) on the right hand side shows distribution of trait under investigation stratified by sex of individuals. DMR window size can be adjusted using the slider bar showing selected number of CpG up- and downstream of CpG under consideration (**e**). For better readability, we do not show real distances between CpG. Distances Information on location, annotation, and effect size is given above the methylation profile plot (**a**)
  The methylation profile plot does not show real distances between probes. It shows only the CpGs available on the microarray in use (in this example, the GPL23976 platform).
(vi) An additional table with all probes shown in the methylation profile plot can be used for exporting methylation data of selected probes together with trait data into external tools (Fig. 7).

**Fig. 7:**
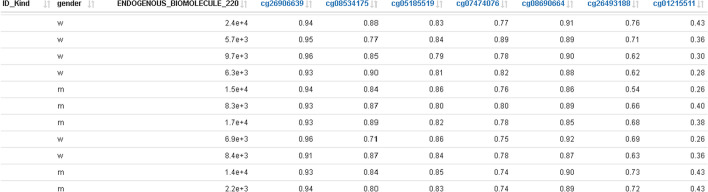
Traits data merged with methylation data; this represents the base data for plotting the methylation profile plot in Fig. 6 together with the selected DMR window and can be used for exporting data to external tools
  The above example (Fig. 3 to Fig. 6) clearly shows a methylation difference of delta methylation = 0.102 (~ 10%) for CpG cg07474076 between the lowest and highest values of the endogenous biomolecule under investigation.
(vii) We furthermore provide a correlation profile plot for selected CpG and its most correlated CpGs of interest (Fig. 8). The table shows correlation coefficients (corr.coeff) together with annotated CpG information (gene.symbol, genomic feature, etc.). Selected CpGs can be visualized in the plot below the table.

**Fig. 8:**
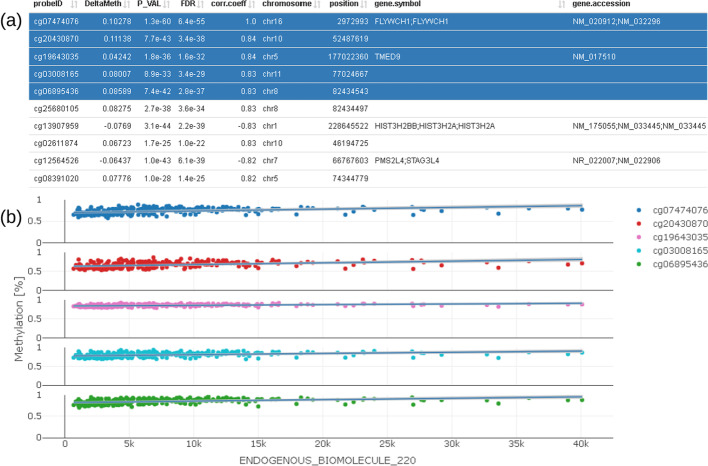
Correlation profile plot (**b**) showing CpG which are correlated to the selected CpG; correlated CpG are selected in the table on the top (**a**) and plotted below using different colors

All these visualization tools should help to obtain a fast impression from prior calculated regression models to make trustworthy conclusions. A detailed description of the workflow and interaction with the software is given in the packages vignette on GitHub.

### Interfacing to external tools

All probeIDs are enriched with additional hyperlinks that link to the EWAS data hub [18] and allow further inspection against prior findings related to this particular probe.

Comparison of standard deviation (SD) at a certain CpG location with findings from other studies can also be used to determine whether a DMP is related with a certain trait under investigation or not. Given a DMP was found in a single cohort and no other cohort with the same trait is available, the SD at this particular probe can be calculated and visualized also without information on the trait under investigation. If the DMP does persist in the compared cohort, then there is a high probability that the DMP was not found by chance.

A further important source for identifying useful associations of DNAm to trait under investigation are annotations leading to pathways, linking together functionally associated and differentially methylated probes at various locations besides correlation. EpiVisR supports the export of probe lists into external tools for pathway analysis by compiling together all necessary data (gene.symbol, p-value, delta methylation) from significant probes. In contrast to gene analysis, where the change in gene expression is measured as an ‘n-fold change’, we use the measure ‘delta methylation’ for results of EWAS data.

### Strengths and limitations

EpiVisR is solely based on R programming language and is based on a local file-system for storage of results, with no local database required. Both were designed this way to ensure high performance. Simultaneously, this is a limitation since interdependencies between different traits under investigation cannot be systematically recognized.

## Conclusions

EpiVisR enables the users to visualize results from regression models, describing methylation profiles for certain trait under investigation. It furthermore allows inspecting the relationship between trait under investigation and methylation in a visual way in order to produce high-quality plots by using the plotly framework and to generate lists of differentially methylated probes for subsequent external analysis (e.g., pathway analysis).

To the best of our knowledge, EpiVisR is the first implementation that covers the aspects of result visualization, enrichment, and linking to external databases for EWAS with multiple trait under investigation. This in turn will allow easier selection of relevant trait under investigation for further inspection.

## Availability and requirements

Project name: EpiVisR. Download location: EpiVisR can be downloaded from https://github.com/SteRoe/EpiVisR. Operating systems: various, platform independent. Programming language: R (version 4.1.2). Additional libraries: *shiny* library (version 1.7.1), *plotly* library (version 4.10.0), *QCEWAS* library (version 1.2–2), *data.table* library (version 1.14.2) [24], *tidyverse* meta package (1.3.1) [36], *RcolorBrewer* library (1.1–2) [37] License: MIT. Restrictions for non-academics use: none.

## Data Availability

A dataset demonstrating the capabilities of the described software is provided among the software itself on https://github.com/SteRoe/EpiVisR.

## Abbreviations

DMP: Differentially methylated CpG probe
DMR: Differentially methylated CpG region
DNAm: CpG methylated DNA
EWAS: Epigenome-Wide Association Studies
SD: Standard deviation
UI: User interface
